# A Long-Term Low-Frequency Hospital Outbreak of KPC-Producing *Klebsiella pneumoniae* Involving Intergenus Plasmid Diffusion and a Persisting Environmental Reservoir

**DOI:** 10.1371/journal.pone.0059015

**Published:** 2013-03-11

**Authors:** Ståle Tofteland, Umaer Naseer, Jan Helge Lislevand, Arnfinn Sundsfjord, Ørjan Samuelsen

**Affiliations:** 1 Department of Clinical Microbiology, Sørlandet Hospital HF, Kristiansand, Norway; 2 Reference Centre for Detection of Antimicrobial Resistance, Department of Microbiology and Infection Control, University Hospital of North Norway, Tromsø, Norway; 3 Research Group for Host-Microbe Interactions, Department of Medical Biology, Faculty of Health Sciences, University of Tromsø, Tromsø, Norway; Amphia Ziekenhuis, The Netherlands

## Abstract

**Background:**

To study the molecular characteristics of a long-term, low frequency outbreak of *bla*_KPC-2_ in a low prevalence setting involving the hospital environment.

**Methodology/Principal Findings:**

KPC-producing bacteria were screened by selective chromogenic agar and Real-Time PCR. The presence of antibiotic resistance genes was ascribed by PCRs and subsequent sequencing, and the KPC-producing isolates were phylogenetically typed using PFGE and multi-locus sequence typing. *Bla*_KPC-2_-plasmids were identified and analysed by S1-nuclease-PFGE hybridization and PCR based replicon typing. A ∼97 kb IncFII plasmid was seen to carry *bla*_KPC-2_ in all of the clinical isolates, in one of the isolates recovered from screened patients (1/136), and in the *Klebsiella pneumoniae* and *Enterobacter asburiae* isolates recovered from the environment (sinks) in one intensive care unit. The *K. pneumoniae* strain ST258 was identified in 6 out of 7 patients. An intergenus spread to *E. asburiae* and an interspecies spread to two different *K. pneumoniae* clones (ST27 and ST461) of the *bla*_KPC-2_ plasmid was discovered. *K. pneumoniae* ST258 and genetically related *E. asburiae* strains were found in isolates of both human and environmental origins.

**Conclusions/Significance:**

We document a clonal transmission of the *K. pneumoniae* ST258 strain, and an intergenus plasmid diffusion of the IncFII plasmid carrying *bla*_KPC-2_ in this outbreak. A major reservoir in the patient population could not be unveiled. However, the identification of a persisting environmental reservoir of strains with molecular determinants linked to human isolates, suggests a possible role of the environment in the maintenance of this long-term outbreak.

## Introduction

The *Klebsiella pneumoniae* carbapenemase (KPC) was first identified in the USA in a *Klebsiella pneumoniae* isolate dated from 1996[1] and has subsequently been reported worldwide.[2] High prevalence rates of KPC have been reported from regions and countries such as USA, Israel, China, and Greece, while a number of countries have reported hospital outbreaks and sporadic cases of KPC-producing *K. pneumonia.* [2]

KPC has been identified in various species of Enterobacteriaceae and non-fermenters such as *Pseudomonas aeruginosa* and *Acinetobacter baumannii.* [2] Further, *bla*_KPC_ has been identified on plasmids differing in size and structure. This broad species distribution and plasmid diversity is likely due to the location of *bla*_KPC_ in a functional Tn*3*-based transposon structure (Tn*4401*) with a high transposition frequency.[3] With respect to *K. pneumoniae*, multilocus sequence typing (MLST) has shown that the global dissemination of KPC-producing *K. pneumoniae* is dominated by isolates belonging to a hyperepidemic clonal complex including sequence type (ST) 258.[4] Although the dissemination and outbreaks of KPC seems to be associated with specific clones several reports are describing outbreaks where several clones and different species are involved [5], [6] as well as individual patients containing different species harbouring *bla*_KPC._ [7], [8], [9]

In many countries the emergence of KPC-producing bacteria has been associated with import of isolates from high prevalent areas. In Norway, the first case of *K. pneumoniae* with KPC was associated with import from Greece in 2007 at a hospital in the southern part of Norway.[10] Subsequently, five additional clinical isolates were identified at the same hospital and a nearby hospital from patients with no recent history of travel or hospitalisation abroad. In this study we describe this long-term outbreak with regards to the molecular characteristics of these isolates, plasmid content and dissemination, and the identification of KPC-producing isolates in the hospital environment.

## Materials and Methods

### Hospital setting

Sørlandet Hospital (SH) is a 683-bed general hospital enterprise located in three different cities in the southern part of Norway. Due to sharing of some specialized medical functions a certain degree of interchange of patients between the hospitals occurs. Two hospitals belonging to Sørlandet Hospital, SH-Arendal (SH-A), and SH-Kristiansand (SH-K), as well as a tertiary hospital (Oslo University Hospital – Rikshospitalet (OUH-RH)) where involved in this outbreak. A screening programme was implemented during outbreak investigation involving a 12-bed (8 single rooms and one 4-bed room) surgical/medical intensive care unit (ICU-A) at SH-A.

### Faecal and environmental screening programme

Faecal screening of ICU-A patients was initiated after the identification of clinical case number 6 (K66-62). Prospective screening was performed on patients (*n* = 50) on admittance and discharge from the ICU-A during a first screening period from 8^th^ of May to 19^th^ of October 2010, and then only on discharge from the ICU-A during a second screening period from 19^th^ of October to 1^st^ of April 2011 (*n* = 65). Retrospective screening was attempted on 29 patients that had been hospitalized in the ICU-A at overlapping intervals with patient 6. Seventeen of these were still hospitalized and screened, whereas 4/12 discharged patients were subjected to screening. Patients staying in the ICU-A for more than 14 days were screened repeatedly every second week.

Screening of the environment was undertaken on two occasions, from the 7^th^ to the 9^th^ of June 2010 and on the 27^th^ of December 2010. The screenings involved sink drains in the ICU-A (n = 19), the neighbouring post-operative unit, the coronary unit, and taps for water to dialysis machines in the ICU-A. Environmental samples were obtained with sterile, cotton-tipped swabs (COPAN swabs*®*). Most areas examined were moist, but the cotton-tip was occasionally moistened when the area examined appeared relatively dry. Screening positive locations were disinfected and then controlled by monthly screening.

### Isolation of KPC-producing bacteria and detection of bla_KPC_ during the screening programme

Screening for KPC-producing bacteria was performed using ChromID ESBL agar medium (bioMerieux, la Balme-les-Grottes, France) and an enrichment medium (tryptic-soy broth containing 2 mg/L cefpodoxime). Real-Time PCR of *bla*_KPC._ was performed directly on suspensions from the swab, enrichment medium, and colonies from the ChromID ESBL agar[11].

### Bacterial strains and clinical data

Clinical data and data on bacterial strains investigated in this study are listed in Table 1 and 2.

**Table 1:** Molecular characteristics of outbreak strains.

Isolate	Source (P/E/F)^ 1^	Month of isolation	Species	PFGE	MLST	*bla*_KPC_^2^	*bla*_SHV_^2^	*bla*_TEM_^2^	Plasmid profiling (kB) ^3^	Plasmid hybridization *bla*_KPC_^4^
K47-25	P1	Nov 07	*K. pneumoniae*	A2	258	KPC-2	SHV-11/-12	TEM-1	40, 97, 160	97,160
K48-58	P2	March 08	*K. pneumoniae*	A2	258	KPC-2	SHV-11/-12	TEM-1	97, 242	97
K52-74	P3	Oct 08	*K. pneumoniae*	A2	ND	KPC-2	SHV-11/-12	TEM-1	40, 97, 194	97
K54-05	P4	Jan 09	*K. pneumoniae*	A2	ND	KPC-2	SHV-11	TEM-1	40, 97/100	97
K57-33	P5	March 09	*K. pneumoniae*	B	461	KPC-2	SHV-1	TEM-1	48, 97, 225	97
K66-62	P6	April 10	*K. pneumoniae*	A1	ND	KPC-2	SHV-11/-12	TEM-1	40, 97, 194	97
K66-73	P7/F	May 10	*K. pneumoniae*	A1	258	KPC-2	SHV-11/-12	TEM-1	40, 97, 194	97
K66-74	P7/F	May 10	*E. asburiae*	C1	-	KPC-2	NEG	TEM-1	97,145	97
K67-04	P1	Jan 10	*K. pneumoniae*	A1	258	NEG	SHV-11/-12	NEG	40, 194	NEG
K67-05	P4	May 09	*K. pneumoniae*	A2	258	NEG	SHV-11/-12	NEG	40, 194	NEG
K67-06	P3	March 10	*K. pneumoniae*	A2	258	NEG	SHV-11/-12	NEG	40, 194	NEG
K67-11	E5	June 10	*K. pneumoniae*	A2	ND	KPC-2	SHV-11/-12	TEM-1	40, 97, 194	97
K67-12	E5	June 10	*E. asburiae*	C2	-	KPC-2	NEG	TEM-1	97,145	97
K67-13	E6	June 10	*K. pneumoniae*	D	27	KPC-2	NEG	TEM-1	45, 97, 200	97
K67-14	E6	June 10	*K. pneumoniae*	D	27	KPC	NEG	TEM-1	45, 97, 200	97
K67-15	E5	June 10	*K. pneumoniae*	A1	ND	KPC	SHV-11/-12	TEM-1	40, 97, 194	97
K67-16	E5	June 10	*E. asburiae*	C2	-	KPC	NEG	TEM-1	97,145	97

1) P1–6 =  clinical specimen from patients 1–6, P7/F =  fecal screen patient 7, E = specimen from environmental screen (rooms 5, 6).

2) *bla*_CTX-M_ was negative in all isolates. *bla*_pAmpC_ was negative in isolates from patients 1–6, others ND.

3) Plasmid profiling (S1-nuclease digested DNA (kB)).

4) Plasmid DNA hybridization with *bla*_KPC_ specific probes.

**Table 2:** Clinical data and risk factors for outbreak patients.

Isolate	Pat-ient	Species	Specimen	Hospita-lization abroad (country)	Month admitted	LOS^6^/LOS prior to diagnosis	Patient overlap^7^	Antibiotic treatment^8^	Other risk factors (I/S/R/D)	Role in infection	Discharged to
K47-25	P1	*K. pneumoniae*	Expectorate	Greece	Nov 07	51/6	Yes3)	MEM, PTZ	I/R	Uncertain	Home
K48-58	P2	*K. pneumoniae*	Urine	No	March 08	20/8	Yes3)	None	None 1)	None	Nursing home
K52-74	P3	*K. pneumoniae*	Blood	No	Sep 08	36/23	No5)	MEM, TOB, CTX	I/R/D	Yes	Physical rehabilitation
K54-05	P4	*K. pneumoniae*	Expectorate	No	Nov 08	106/47	Yes^4,^ 5)	MEM,TOB	I/R/D	Uncertain	Physical rehabilitation
K57-33	P5	*K. pneumoniae*	Urine	No	Nov 08	178/131	Yes4)	MEM, IMI	I/S/R	Uncertain	Nursing home
K66-62	P6	*K. pneumoniae*	Expectorate	No	April 10	24†/17	No	MEM	I/S/R	None	Diseased (in hospital)
K66-73 K66-74	P7	*K. pneumoniae E. asburiae*	Feces/fecal screening	No	May 10	13/5	No	CTX	I/R	None	Home

1) Except urinary catheter.

2) I =  admission to ICU, S =  recent surgery (laparotomi), R =  artificial ventilator use, D =  subjected to haemodialysis.

3) Patient 1, being readmitted to SH-K, and patient 2 had been hospitalized in the same corridor for two days in March 2008 at SH-K although at separately staffed wards (Med. 2A-K and Med. 2C-K). Measures for contact isolation were not implemented for patient 1 on this occasion.

4) Patient 4 and patient 5 had been admitted simultaneously to the ICU-A, and had also been referred to a tertiary hospital (OUS-RH) at overlapping intervals.

5) Patient 3 and patient 4 were hospitalized in the ICU-A two days apart.

6) LOS = length of stay (days).

7) Overlap between patients in time and wards.

8) Anti G-negative antibiotics prior to diagnosis

### Species identification

Bacterial identification was performed using the VITEK2 ID-GNB system (bioMérieux, Marcy l'Etoile, France) and/or MALDI-TOF (Microflex LT, Bruker Daltonics) with the MALDI Biotyper 3.0 software version.

### Antimicrobial susceptibility testing

Antimicrobial susceptibility of the isolates was determined using Etest (bioMérieux) according to the manufacturer's instruction. The results were interpreted using breakpoints from the European Committee for Antimicrobial Susceptibility Testing.[12]

### Molecular characterisation of KPC-producing isolates

PCR and sequencing for *bla*_KPC_, *bla*_TEM_, *bla*_SHV_, *bla*_CTX-M_, and a multiplex PCR for the most prevalent plasmid-borne *bla*_AmpC_ -genes was performed as previously described (Samuelsen JAC 09). Isolates were typed by pulsed-field gel electrophoresis (PFGE) of *Xba*I-digested total genomic DNA.[10] Strain relatedness was analysed using BioNumerics version 6.0 (Applied Maths, Sint-Martens-Latem, Belgium) using the band-based dice coefficient and the unweighted pairs geometric-matched analysis dendrogram with a position tolerance of 1% for optimization and band comparison. Final comparison of bands for the interpretation of relatedness was performed in accordance with Tenover criteria.[13] Multi-locus sequence typing (MLST) was performed on *K. pneumoniae* isolates according to the protocol described on the *K. pneumoniae* MLST web site http://www.pasteur.fr/recherche/genopole/PF8/mlst/Kpneumoniae.html.

### Plasmid analysis

Plasmid profiling was performed using S1-nuclease digested plasmid DNA, separated by PFGE.[10] Subsequently, plasmid DNA was blotted onto nylon membrane and hybridised with *bla*_KPC_ specific probes to identify carrier plasmids. Furthermore, plasmids were classified into incompatibility groups using the PCR based replicon-typing (PBRT) scheme of Carattoli[14] and hybridisation with replicon FII specific probes as previously described.[10]

### Transfer of resistance

Conjugal-transfer experiment was carried out in Luria-Bertani broth at 37°C for clinical strain K47-25 as donor and rifampicin-resistant E. coli J53-2 as recipient, mixed in a 1∶9 ratio respectively. Transconjugants were selected on LB-agar plates (Becton Dickinson, Sparks, MD) supplemented with 100 mg/L rifampicin (Sigma-Aldrich, St. Louis, MO) and 2 mg/L ceftazidime (Sigma-Aldrich).

### Ethics statement

This study focuses on the molecular characteristics of bacterial isolates collected as part of the clinical management and microbiology routine work. Faecal screening was performed according to the guidelines from the local hospital and collection of clinical data as part of outbreak investigations for implementation of appropriate infection control measurements. Consequently, ethical approval was not obtained for the study.

## Results

### Clinical cases

Six KPC-producing *K. pneumoniae* isolates from clinical samples were recovered in SH from November 2007 to April 2010 (Table 1 and Table 2). The first two isolates had been characterised previously.[10] The first patient had been hospitalized on Crete in September 2007 prior to hospital admission in Norway. The other patients had no history of recent travel abroad. 5 isolates were recovered from SH-A, whereas the isolate from patient 2 was recovered from SH-K. After the identification of KPC-producing *K. pneumoniae*, measures for contact isolation were initiated for all patients. When patient 1 was readmitted at SH-K in March 2008 measurements for contact isolation was however not implemented. Retrospective analysis of patient histories revealed that all SH-A patients had been hospitalized in the same ICU (ICU-A) as the index patient prior to detection of the KPC-producing isolates. However, during their stay the patients had been admitted to 12 different wards. Further, conventional epidemiological evaluation[15] was able to establish epidemiological links between patient 1 and 2, and 3 and 4, while a possible link was suggested between patient 4 and 5 (Table 2). Time and space overlaps between the clinical cases 1 and 3 as well as 5 and 6 could not be provided for, representing time intervals of 10 and 11 months, respectively.

Several recognized risk factors for ESBL colonization and infection[15], [16], [17], [18] were present in all the patients that had been admitted to the ICU-A prior to isolation of KPC-producing *K. pneumoniae* (patient 1, 3–6) (Table 2). All patients were above 65 years of age. KPC-producing *K. pneumoniae* bacteraemia was recognized in one patient, whereas the findings in the other patients were regarded as most likely to be colonisations associated with artificial ventilation or urinary catheter. One patient died, but the KPC-producing *K. pneumoniae* was not considered to be attributable to infection or death in this patient (Table 2).

ESBL-producing *bla*_KPC_-negative *K. pneumoniae* were also recovered in ambulatory urinary samples due to urinary tract infections (UTI) from three of the of the patients 26, 16, and 4 months after the initial isolation of the KPC-producing *K. pneumoniae* isolates, respectively (Table 1).

### Patient screening

In the patient screening programme only 1/136 patients were positive for KPC-producing Enterobacteriaceae (patient 7). Patient 7, being hospitalized in room 5 for his entire ICU-A stay, was identified with KPC-producing *K. pneumoniae* (K66-73) and *Enterobacter asburiae* (K66-74) upon discharge (Table 1), while negative upon admission five days earlier. Room 5 was disinfected using a persulfate based disinfectant after having been occupied by patient 6 and then patient 7 was the first to be admitted two days later.

### Environmental screening

KPC-producing bacteria were detected in 4/19 environmental locations in the ICU-A (sink drains in room 5, 6, 9, and the rinsing room). *Bla*_KPC_-positive *K. pneumoniae* were identified in June 2010 from the sink drains in room 5 (K67-11 and K67-15) and room 6 (K67-13 and K63-14), and *bla*_KPC_-positive *E. asburiae* from the sink drain in room 5 (K67-12 and K67-16) (Table 1). The sinks and sink traps were decommissioned and the connecting pipe elbows were disinfected using a chlorine disinfectant before new sinks and sink traps were installed. Monthly environmental screening of these positive locations was then undertaken. *Bla*_KPC_-positive *K. pneumoniae* was again recovered from the sink in room 6 on two occasions in December 2010 triggering a new environmental screening in the ICU-A (data not shown). Additional sink drains in the ICU-A was investigated, and two new locations that were not subjected to investigation in June 2010 were now identified with *bla*_KPC_-positive *K. pneumoniae* (room 9) and *bla*_KPC_-positive *E. asburiae* (room 9 and the rinsing room, data not shown).

Although *bla*_KPC_-positive isolates have been identified in the environment no additional clinical cases has been identified since patient 6.

### Molecular characterisation of the isolates

**Figure 1:**
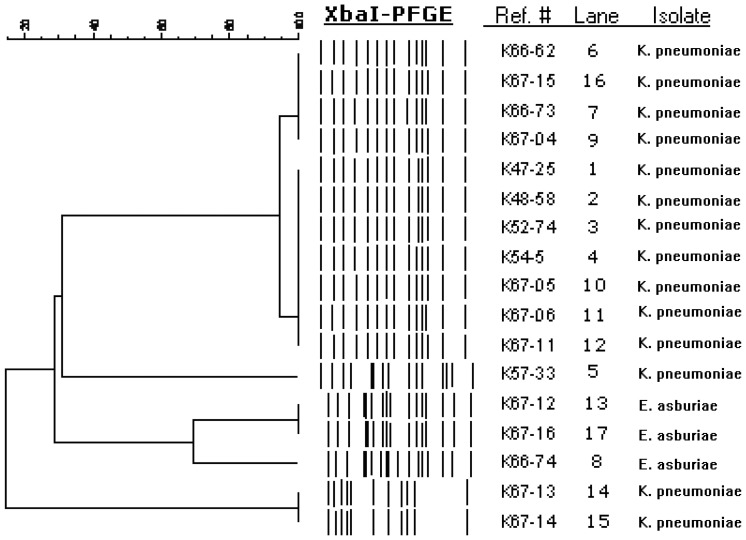
Dendrogram of XbaI-digested genomic DNA. Strains of KPC-producing *K. pneumoniae-* and *E.asburiae*-isolates and ESBL_A_-producing *K. pneumonia* are shown with percentages of similarity to the right of the dendrogram.

PFGE and MLST typing revealed that 14 *K. pneumoniae* isolates from both patients and the environment, including the three *bla*_KPC_-negative *K. pneumoniae* UTI-isolates, belonged to two clonally related pulsotypes (A1 and A2), that by MLST were typed to ST258 (Figure 1, Table 1). Distinct *K. pneumoniae* PFGE/MLST-types were recovered in patient 5 (K57-33; pulsotype B/ST461) and isolates from the sink drain in room 6 (K67-13 and K67-14; pulsotype D/ST27). PFGE of the *E. asburiae* isolates revealed two closely related pulsotypes corresponding to the *E. asburiae* (pulsotype C1) isolate from the screening patient (patient 7; K66-47) and the two *E. asburiae* (pulsotype C2) isolate from room 5 (K67-12 and K67-16). The *bla*_KPC_-gene was sequenced in all isolates from patients and from the environmental in June 2010 and found to be *bla*_KPC-2_ (Table 1). The *bla*_KPC_-positive *K. pneumoniae* ST258 isolates harboured *bla*_SHV-11_, *bla*_SHV-12_, and *bla*_TEM-1_, whereas the three *bla*_KPC_-negative *K. pneumoniae* ST258 UTI-isolates, harboured *bla*_SHV-12_ and *bla*_SHV-11_, but were negative for *bla*_TEM_. The distinct *K. pneumoniae* STs in patient 5 (K57-33) and from room 6 (K67-13 and K67-14) harboured *bla*_SHV-1_ and lacked the *bla*_SHV_-gene, respectively. The *bla*_KPC_-positive *E. asburiae* isolates were devoid of *bla*_SHV_ but harboured *bla*_TEM-1_ (Table 1). PCRs for *bla*_CTX-M_ and the most prevalent plasmid-borne *bla*_AmpC_ genes were negative.

### Plasmid analysis

**Figure 2:**
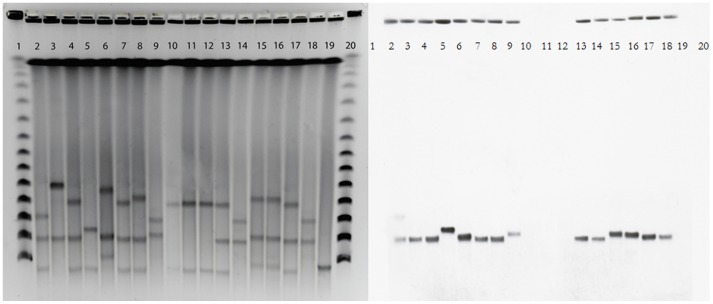
PFGE of S1 nuclease-digested total DNA. Lanes 1 and 20, phage λ DNA ladder (concatemers of 48.5 kb); lane 2, K47-25; lane 3, K48-58; lane 4, K52-74; lane 5, K54-05; lane 6, K57-33; lane 7, K66-62; lane 8, K66-73; lane 9, K66-74; lane 10, K67-04; lane 11, K67-05; lane 12, K67-06; lane 13, K67-11; lane 14, K67-12; lane 15, K67-13; lane 16, K67-14; lane 17, K67-15; lane 18, K67-16; lane 19, *K. pneumoniae bla*_KPC_ -negative control strain.

S1-nuclease-PFGE profiles revealed the presence 2-3 plasmids in all isolates ranging from∼40–∼240 kb (Table 1, Figure 2). Hybridization with *bla*_KPC_-specific probes identified a *bla*_KPC_-carrier plasmid to approximately 97 kb in all *bla*_KPC_-positive isolates of *K. pneumoniae* and *E. asburiae*. In the *bla*_KPC_-positive *K. pneumoniae* isolate from patient 1 (K47-25) an additional *bla*_KPC_-plasmid of ∼160 kb was identified. PBRT and hybridization characterized the ∼97 kb plasmid as an IncFII replicon plasmid. Except for the 97 kb *bla*_KPC_-plasmid, separate plasmid profiles were recognized in the other *K. pneumoniae* pulsotypes/STs and the closely related *E. asburiae* clones. The plasmid profile among the *E. asburiae* isolates were identical (Table 1, Figure 2).

Interestingly, the plasmid profile of the pulsotype A/ST258 *bla*_KPC_-negative *K. pneumoniae* clinical UTI isolates was devoid of the ∼97 kb *bla*_KPC_-plasmid, indicating loss of this plasmid (Table 1).

### Transferability of bla_KPC_ resistance

*In vitro* plasmid-transfer experiments using K47-25 as the donor strain to *E. coli* resulted in transconjugants with a frequency of 2.5×10^−7^ per donor cells.

### Antimicrobial susceptibility

The antimicrobial susceptibility profile of the isolates is shown in Table 3. Variable levels of reduced susceptibility to carbapenems were observed in all the *bla*_KPC_-positive isolates whereas the three ESBL-positive *bla*_KPC_-negative *K. pneumoniae* ST258 UTI isolates were susceptible to carbapenems (Table 1). A similar susceptibility profile with high-level resistance to penicillins and cephalosporins, resistance to ciprofloxacin, tobramycin, and amikacin, but susceptibility to gentamicin, were common to all *K. pneumoniae* ST258/pulsotype A isolates. In contrast, the other *bla*_KPC_-positive *K. pneumoniae* pulsotypes were susceptible to ciprofloxacin, tobramycin, amikacin, and the gentamicin MIC was lower. The *bla*_KPC_-positive *E. asburiae* isolates found in the screening positive patient (patient 7) and the environment showed resistance to ciprofloxacin, but were susceptible to the aminoglycosides.

All *bla*_KPC_-positive *K. pneumoniae* ST258 isolates were resistant to colistin. 2 of 3 *bla*_KPC_-negative *K. pneumoniae* UTI isolates were susceptible to colistin, whereas one isolate was resistant (K67-05) displaying a clearly visible double zone (MIC 0.5 mg/L and MIC 16 mg/L).

## Discussion

The dissemination of multidrug resistance plasmids among Gram-negative bacteria is one of the major factors contributing to the spread of antimicrobial resistance.[19] However, information about plasmid-dissemination during outbreaks is relatively limited. Often the focus is restricted to specific bacterial species and the dissemination of plasmids into different species might be overlooked. Highly mobile plasmids resulting in ‘plasmid-borne outbreaks’ may delay the recognition of an outbreak, and adds an additional layer of complexity to the molecular investigation of such outbreaks.[20] Plasmids have previously been linked to outbreaks of multi-drug resistant (MDR) Gram-negative bacteria[21], [22], [23], [24], [25], including nosocomial outbreaks of *bla_KPC_*-containing plasmids in some studies.[5], [7], [8], [20], [26], [27], [28], [29] Here we describe the dissemination of a KPC-plasmid between strains and species during a small long-term nosocomial outbreak in a low-prevalence setting as well as the possible role of the environment in this context. To the best of our knowledge this is the first nosocomial outbreak report of KPC-producing bacteria from the Nordic countries.

I*n vivo* mobility is indicated by identification of the ∼97 kb IncFII *bla*_KPC-2_-plasmid in four distinct MLST/PFGE types of *K. pneumoniae* and *E. asburiae.* This observation was supported by successful *in vitro* conjugation of *bla*_KPC_-plasmids. *bla*_KPC_ –containing Tn*4401* has been documented on plasmids of different sizes belonging to different incompatibility groups.[30], [31], [32], [33], [34] While mostly being reported on broad host IncN plasmids[31], *bla*_KPC_ has recently been reported on narrow host range IncFII plasmids as well, promoting efficient spread within members of Enterobacteriaceae.[33], [35], [36]

It is well known that plasmid-transfer can occur *in vivo,* but less is known about plasmid-transfer occurring in the hospital environment. We can only speculate were the plasmid-transfer has occurred in this setting. The first clinical cases were associated with the hyperepidemic *K. pneumoniae* ST258 strain before plasmid-dissemination were observed into other *K. pneumoniae* STs (ST27 and ST461) and *E. asburiae*, all of them devoid of non-β-lactam resistances except the latter displaying low-level ciprofloxacin resistance. The KPC-producing *E. asburiae* remained clinically silent throughout the outbreak and first appeared in faecal screening of patient 7. Faecal screening was not performed on any of the clinical patients, thus whether KPC-producing *E. asburiae* was carried by any of these patients remains elusive. The finding of a distinct *K. pneumoniae* (ST27) with the ∼97 kb KPC-plasmid in the hospital environment but not in patients could either be due to the unrecognized presence in patients and *in vivo* plasmid-transfer or that the plasmid-transfer have occurred in the environment before acquisition by a patient.

Interestingly, loss of *bla*_KPC_-plasmids, ∼160 kb plasmid in patient 1 and the ∼97 kb plasmid in the three *bla*_KPC_-negative *K. pneumoniae* UTI isolates, was observed in *K. pneumoniae* ST258 indicating either a high fitness cost or reduced plasmid stability. Experiments to determine the fitness cost related to the ∼97 kb plasmid and its stability are ongoing.

Reservoirs in the patient or health care worker populations and the environment represent principle modes of spread in nosocomial outbreaks with the patient population being the most important reservoir in high-frequent outbreaks.[37] Introduction of the *K. pneumoniae* ST258 clone has caused major outbreaks in many hospitals.[38] It is possible that a high level of adherence to standard precautions prevented the establishment of a major gastrointestinal reservoir in our patients, and thus, the prerequisite of a major high-frequent outbreak, were not present.[37], [39], [40], [41] The lack of a patient reservoir and the persistence of the outbreak despite the long interval between known clinical cases could indicate other possible reservoirs contributing to occasional transmissions and maintenance of the outbreak. Health care workers were not screened for faecal or hand carriage partly as no individuals with additional risk factors including dermatitis[37] could be identified and partly due to the fact that the current Norwegian health authority guidelines advocates against screening of health care workers in nosocomial outbreaks of multidrug resistant Gram-negative bacteria.[42] KPC-producing bacteria were detected in as much as 21% of environmental locations in screening samples from sink drains in the ICU-A. Moist surfaces and especially sink drains have been focused in several studies as a possible environmental source of transmission of Enterobacteriaceae, especially *Klebsiella* spp, to patients.[43], [44], [45], [46], [47], [48] Although the environment were decontaminated and sinks were replaced, KPC-producing *E. asburiae* and *K. pneumoniae* were recovered during further environmental screening, suggesting that these strains can survive well in that environment. Contamination of the hands of health care workers due to occasional backsplash during hand washing in a contaminated sink and sink drains[44], [46], [47], [49] or through moist surfaces near sinks and faucets[45] has been suggested as a possible mode of transmission to health care workers and subsequently to patients in the ICU setting, facilitating low-frequent transmissions. The epidemiological link between patient 6 and 7 through their succeeding use of room 5 and the isolation of pulsotype identical KPC-producing *K. pneumoniae* ST258 from both patients and the sink drain in that room as well as the recovery of closely related KPC-producing *E. asburiae* from faecal screening in patient 7 and the sink drain could suggest a possible environmental source of colonization of patient 7.

Investigation revealed that several sinks were heavily contaminated by bacteria presumably from waste water washed down the sink after cleaning of patients. A ‘bed bath’-system for cleaning of patients were implemented to shortcut this opportunity. Repetitive disinfection with a biofilm active disinfectant was considered, but disinfection was only performed on two occasions; in June 2010 and January 2011.

In conclusion; the outbreak involved clonal spread of the hyperepidemic *K. pneumoniae* ST258 stain as well as interspecies plasmid transmission of a ∼97 kb *bla_KPC_*-plasmid into two other distinct *K. pneumoniae* strains and intergenus spread to *E. asburiae*. The establishment of a local environmental reservoir was documented. The spread among patients has probably occurred partly as a result of transmission between patients in some of the clinical cases, but we infer the possibility that the outbreak was maintained and prolonged with additional clinical cases added due to spread from environmental sources.
